# Toward the rational design of oncogenic TASK-3 channel inhibitor peptides and nanoconjugate complexes

**DOI:** 10.3389/fphar.2026.1807764

**Published:** 2026-05-29

**Authors:** Leandro Zúñiga, Wendy González, Rafael Zúñiga, Whitney Venturini, Cristian Vilos

**Affiliations:** 1 Laboratorio de Fisiología Molecular, Facultad de Medicina, Universidad de Talca, Talca, Chile; 2 Center for Nanomedicine, Diagnostic, and Drug Development (ND3), Facultad de Medicina, Universidad de Talca, Talca, Chile; 3 Center for the Development of Nanoscience and Nanotechnology (CEDENNA), Proyecto CIA250002, Facultad de Medicina, Universidad de Talca, Talca, Chile; 4 Centro de Bioinformática y Simulación Molecular, Universidad de Talca, Talca, Chile; 5 Departamento de Medicina Traslacional, Facultad de Medicina, Universidad Católica del Maule, Talca, Chile; 6 Laboratory of Nanomedicine and Targeted Delivery, School of Medicine, Universidad de Talca, Talca, Chile

**Keywords:** cancer, ion channels, nanoconjugate, nanomedicine, peptides, phage display, targeted therapy, TASK-3

## Abstract

TASK-3 potassium channels have emerged as important regulators in several aspects of cancer pathophysiology. Overexpression of TASK-3 occurs in a broad spectrum of cancers, including breast, lung, ovarian, colorectal cancers, and melanoma. Genetic or pharmacological inhibition of TASK-3 has been shown to suppress tumorigenic properties. Despite the strong evidence, the development of selective TASK-3 inhibitors remains limited. In this review, we analyze the current evidence supporting TASK-3 as an oncogenic target, with emphasis on peptide-based inhibitors and advanced delivery strategies. Also, we review the landscape of TASK-3 modulation across different cancer types, summarize known mechanisms of ion channel inhibition, and highlight the advantages of peptides for achieving target selectivity and specificity. We further explore nanoconjugate delivery systems to improve peptide stability, bioavailability, and tumor targeting. Finally, we outline rational design methods, phage display technologies, and electrophysiological validation as an integrated pipeline for developing next-generation TASK-3 inhibitors. Together, these approaches delineate a strategic and technical framework for advancing selective TASK-3–targeted therapeutics in oncology.

## 1 Introduction

Ion channels are integral membrane proteins that facilitate the flux of ions across cellular membranes and play essential roles in cell signaling, proliferation, metabolism, and survival. These proteins have emerged as critical players in the development and progression of cancer (Zúñiga et al., 2022). Particularly, the K2P (two-pore domain potassium channel) family has garnered significant attention in cancer research (Lotshaw, 2007; Huang and Jan, 2014; Zúñiga et al., 2022). Within this family, TASK-3 channel (K2P9.1), has been recognized for its oncogenic properties. TASK-3 dysregulation actively contributes to the acquisition of cancer hallmarks (Zúñiga et al., 2022), positioning TASK-3 as a promising target for the development of novel cancer therapeutics. TASK-3 is overexpressed in a wide range of cancers, including breast, lung, ovarian, colorectal, and melanoma (Zúñiga et al., 2022). Gain-of-function of TASK-3 has been associated with increased resistance to hypoxia and serum deprivation (Zúñiga et al., 2018). Reducing TASK-3 expression via knockdown has been shown to inhibit cancer cell growth and even induce cellular senescence (Zúñiga et al., 2018). These observations strongly suggest that TASK-3 plays a crucial role in tumorigenesis and its inhibition could offer a viable strategy for cancer treatment.

However, developing highly specific inhibitors remains a challenge, particularly given the high homology of TASK-3 within the K2P ion channel family (Goldstein et al., 2001; Lotshaw, 2007; Bittner et al., 2010; Zúñiga and Zúñiga, 2016). Structural and functional evidence indicate that discrete regions of TASK-3 contribute to selective ligand recognition. In particular, the Cap domain and the extracellular ion pathway (EIP) constitute accessible and potentially targetable interfaces (González et al., 2013; Zúñiga and Zúñiga, 2016). A key determinant of subtype selectivity resides at position 70 within the EIP, where TASK-3 contains a glutamate (E70) and TASK-1 a lysine (K70). This substitution alters the local electrostatic environment and critically governs sensitivity to Ruthenium Red (Czirják and Enyedi, 2003), supporting its role as an electrostatic hotspot for ligand discrimination. In addition, lateral fenestrations connecting the lipid membrane to the central cavity have been structurally identified as alternative access pathways and binding sites for small molecules, further contributing to subtype-specific pharmacology (Dong et al., 2015).

In this regards, peptide-based therapeutics offer several potential advantages. They can be designed to exhibit high specificity and affinity for their targets, potentially minimizing off-target effects (Ramírez et al., 2019b). Unlike small molecules, peptide inhibitors support to address some disease targets, which are difficult to treat with small molecules (Wang et al., 2021). Small molecules frequently exhibit undesirable selectivity, leading to off-target side effects (Lin et al., 2019). Although small molecules have proven to be highly effective, and much is now known about their mechanism of action, they often exhibit cross-reactivity within the TASK family. Also, in many cases, these activate other classes of K^+^ channel, thereby limiting their potential use (Schewe et al., 2019; Rödström et al., 2024). Moreover, target proteins often undergo conformational changes that alter the shape and properties of their interaction surfaces, creating dynamic interfaces (Baeza et al., 2025). Small molecules, due to their rigid structures, struggle to adapt to these variations. In contrast, peptides’ flexible backbones allow them to adapt to surface changes, establishing stronger and more stable contacts (Trisciuzzi et al., 2023; Baeza et al., 2025). Furthermore, peptides can be engineered to target specific protein-protein interaction interfaces (Wang et al., 2021), and mimic key structural domains in target proteins, with increased bioavailability and improved permeability. Additionally, peptides may exhibit a more specific, less invasive, and lower immunogenicity than larger protein-based therapeutics, such as antibodies (Wang et al., 2021). Many antibody-based therapies are currently used in clinical practice for treatment of various disorders and offer several potential benefits compared to traditional approaches, although their identification and development can be costly (Zhu et al., 2022; Rödström et al., 2024). Given the high specific expression of some K^+^ channels in tumors and their tumor-promoting effect, antibodies that specifically bind to K^+^ channels can facilitate the efficient recognition of tumor cells (Xia et al., 2023). However, antibody therapies face challenges such as limited solubility, immunogenicity, and their highly specific targeting nature.

Peptides can target cancer cells, unlike most currently used chemotherapeutic agents. Due to their amphipathic properties, peptides can selectively seek out and bind to cancer cells compared to normal cells because of an aberrant electrical charge difference in the cell surface bilayer of cancer cells (Mizejewski, 2024). They can bind and enter to the cancer cells, causing cell cycle arrest (Mizejewski, 2024). Furthermore, peptides are ideal for use as postoperative oncology agents in combination with standard chemotherapy drugs; this adjuvant action could prevent tumor regrowth after surgical excision (Mizejewski, 2024). Therefore, the design of inhibitory peptides targeting specific regions of TASK-3 channel presents a compelling avenue for developing more selective and effective cancer therapies.

In recent years, nanoconjugate peptide delivery systems have emerged as a promising strategy to enhance the potential of inhibitory peptides (Chavda et al., 2022). These systems can improve stability, protect against enzymatic degradation, and enhance bioavailability. By functionalizing nanoparticles, with targeting peptides, it is possible to achieve the inhibitor delivery directly to the tumor microenvironment increasing efficacy and reducing systemic toxicity (Raha et al., 2011). Thus, the rationally designed peptide inhibitors combined with nanoconjugate delivery systems holds significant promise for revolutionizing the treatment of cancers driven by TASK-3 channel activity.

## 2 The role of TASK-3 channels in cancer

**FIGURE 1:**
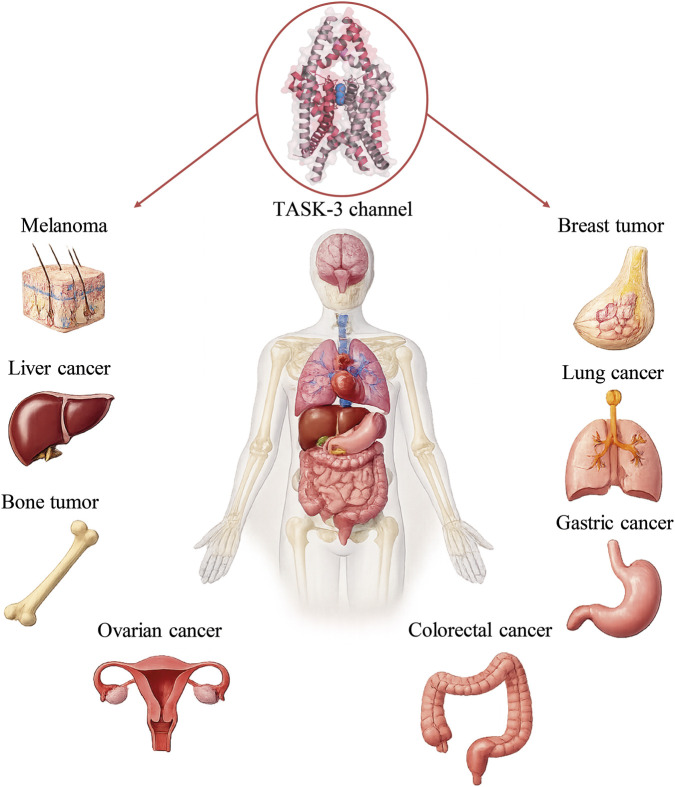
A schematic representation of the human body highlights TASK-3 ion channel expression in cancers of different histogenesis.

The involvement of TASK-3 channels in various malignancies (Figure 1) has been extensively documented. In human breast cancer, TASK-3 is overexpressed in up to 44% of cases (Mu et al., 2003), and in established cell lines such as MDA-MB-231 and MCF-10F (Zúñiga et al., 2018). Downregulation of TASK-3 in MDA-MB-231 cells leads to a significant reduction in cell proliferation, induction of cellular senescence, and cell cycle arrest (Zúñiga et al., 2018). Furthermore, overexpression of the dominant-negative TASK-3/G95E mutation disrupts its oncogenic functions in breast cancer cells (Pei et al., 2003). Notably, inhibiting mitochondrial TASK-3 with a targeted inhibitor (mitoIN-THPP) has been shown to decrease breast cancer cell survival (Bachmann et al., 2021).

Similar to breast cancer, lung cancer also exhibits frequent TASK-3 overexpression up to 35% (Mu et al., 2003). Research indicates that inhibiting TASK channels can reduce cell proliferation in small cell lung cancer (SCLC) (Bittner et al., 2010), reduces cell viability and increases cell death of non-small cell lung cancer (NSCLC) (Leithner et al., 2016; Sun et al., 2016). This suggests that TASK-3 contributes to the pathogenesis of lung cancer.

In ovarian carcinoma, the incidence of TASK-3 overexpression is particularly pronounced, with more than 90% of tumors exhibiting elevated channel levels (Innamaa et al., 2013). Furthermore, TASK-3 has been reported to be overexpressed in ovarian cancer cells (Hu et al., 2024). Paradoxically, heightened TASK-3 expression in this context has been associated with improved patient survival (Innamaa et al., 2013). Nevertheless, pharmacological blockade of TASK-3 with zinc and methanandamide has been shown to markedly suppress cellular proliferation and promote apoptotic cell death in established ovarian cancer cell lines SKOV-3 and OVCAR-3 (Innamaa et al., 2013).

TASK-3 overexpression has also been documented in gastric, colorectal, and melanoma cancers (Kim et al., 2004; Kovács et al., 2005; Pocsai et al., 2006; Rusznák et al., 2008; Toczylowska-Maminska et al., 2014; Cikutović-Molina et al., 2019). Notably, the mitochondrial TASK-3 inhibitor mitoIN-THPP has been shown to kill melanoma cell lines efficiently (Bachmann et al., 2021). These findings emphasize the involvement of TASK-3 channels across a spectrum of human malignancies, suggesting that targeting this channel could have widespread therapeutic implications in oncology.

The Table 1 summarizes the cancer types where TASK-3 has been implicated and the observed effects of its modulation.

**TABLE 1:** TASK-3 channel and their impact in cancer.

Tumor or cancer type	Expression/Activity	Effects of TASK-3 overexpression	Effects of TASK-3 Inhibition/Downregulation	References
Breast tumors	++	Modulation of cell cycle, proliferation and survival, resistance to hypoxia/serum deprivation	Reduced proliferation, induction of senescence and cell cycle arrest, abrogation of oncogenic functions by mutation	Dookeran et al. (2017), Zúñiga et al. (2018)
Lung cancer	++	Promotes proliferation, cell viability, tumor growth, lung colonization	Decreased cell proliferation	Mu et al. (2003), Pei et al. (2003), Leithner et al. (2016), Sun et al. (2016)
Liver cancer	++	Proliferation, invasion and metastasis of	ND	Li et al. (2019)
Gastric cancer	++	Proliferation, migration and invasion	Reduced of cell proliferation and viability, cell migration and invasion, and increase apoptosis	Cikutović-Molina et al. (2019)
Ovarian cancer	+	Confers survival advantage	Reduced of cell proliferation and increase apoptosis	Innamaa et al. (2013), Hu et al. (2024)
Colorectal Cancer	++	Modulation of cell cycle and proliferation	ND	Kim et al. (2004)
Melanoma	++	Promote proliferation and/or survival of malignantly transformed cells, migration and prevent apoptosis	Decreased survival and viability, and increased rate of apoptotic activity	Pocsai et al. (2006), Rusznák et al. (2008), Kosztka et al. (2011), Nagy et al. (2014)
Bone tumor	+	Cell proliferation and tumorigenesis	Downregulated cell proliferation	Li et al. (2013)

+ = expressed, ++ = overexpressed, ND, not determined

## 3 Current landscape of peptide inhibitors targeting TASK-3 channels

Despite compelling evidence for TASK-3’s role in cancer, developing of highly selective and potent inhibitors for this channel has been challenging (Bittner et al., 2010; Zúñiga et al., 2022). Although highly selective peptide inhibitors for TASK-3 in cancer remain an active area of research, several insights into the molecular mechanisms of existing TASK-3 modulators provide clues for rational design (Decher et al., 2021). Some inhibitors act by directly occluding the channel pore, thereby preventing ion flux (Decher et al., 2021). For example, PK-THPP, a potent TASK-3 antagonist, has been shown to bind to the channel when its fenestrations are open, via hydrophobic contacts with residues in the binding site (Ramírez et al., 2019a). Similarly, breathing stimulant compounds such as PK-THPP and A1899 are believed to inhibit the TASK-3 channel by binding within the channel’s intracellular pore region (Chokshi et al., 2015).

TASK-3 is also modulated by various physiological factors. Extracellular acidification inhibits TASK-3 channels. This pH sensitivity is conferred by H98 near the GYG sequence (Kim et al., 2000; Rajan et al., 2000; Lopes et al., 2001). Additionally, the anandamide has been shown to inhibit both TASK-1 and TASK-3 channels (Zhang et al., 2009). These diverse mechanisms of modulation suggest multiple potential sites on the TASK-3 channel that could be targeted by inhibitory peptides.

A novel TASK-3 inhibitor identified through virtual screening (compound 1) exhibited micromolar inhibitory activity (IC_50_ = 14.2 μM) (Ramírez et al., 2019b), and subsequent structural modifications led to a 4-fold increase in potency (Ramírez et al., 2019b). Furthermore, the mitochondriotropic TASK-3 inhibitor mitoIN-THPP has demonstrated efficacy in decreasing breast cancer cell survival and inducing cell death in melanoma lines (Bachmann et al., 2021). In ovarian cancer cell lines, TASK-3 channel blockers such as zinc and methanandamide have significantly inhibited cell proliferation and increased apoptosis (Innamaa et al., 2013).

Identifying highly selective small-molecule drugs targeting the K2P subfamily has been complex due to the high degree of structural and functional conservation not only among K2P channels but also within the K^+^ channel superfamily. Consequently, the identification of highly selective small-molecule agonists and/or antagonists that exhibit no cross-reactivity with other K^+^ channels is a challenge (Bagal et al., 2013; Schewe et al., 2019). Although small molecules have shown to be highly effective agonists, and much is now known about their mechanism of action, they often exhibit cross-reactivity within the K2P subfamily. Also, in many cases, these agonists activate other classes of K^+^ channel thereby limiting their potential use (Schewe et al., 2019). Other approaches to target these channels may therefore be required.

For some classes of ion channels, channel-adapting toxins and the development of RNA-based antagonists have been successful in achieving greater target selectivity (Zhao Y. et al., 2015; Huang and Niu, 2019), but the most promising approaches involve biological products that exploit protein-protein interactions for target recognition (Tiede et al., 2017; Wulff et al., 2019; Colecraft and Trimmer, 2022; Selvakumar et al., 2022). In this regard, peptides can exhibit greater stability and solubility than conventional antibodies, so their relative ease of production and modification makes them ideal candidates for therapeutic strategies where small-molecule approaches have stalled (Steeland et al., 2016). Furthermore, reports with nanobodies also highlight mechanistic approaches for the study of K2P channels and their function *in vivo*, and expand the potential of nanobodies as therapeutic agents (Rödström et al., 2024). Although the provided research material does not detail existing inhibitory peptides specifically targeting TASK-3, the success of peptides in modulating other K2P channels, such as spadin’s blockade of TREK-1 with antidepressant effects (Moha ou Maati et al., 2016), underscores the feasibility of this approach.

## 4 Nanoconjugate complexes for enhanced delivery of therapeutic peptides

Nanotechnology is a transformative field in cancer therapeutics, offering innovative solutions to the limitations of drug delivery (Yao et al., 2020). Nanoparticle-based delivery systems present numerous advantages for cancer treatment (Chavda et al., 2022). These advantages include improved stability of peptides, enhanced biocompatibility, and targeted delivery, thereby minimizing off-target effects (Kim and Park, 2024). The enhanced permeability and retention (EPR) effect, in which nanoparticles preferentially accumulate in tumor tissues, further enhances the efficacy of nanomedicine in cancer research (Kim and Park, 2024). In the context of TASK-3-driven cancers, these advantages are particularly relevant given the plasma membrane localization and extracellular accessibility of the channel, which enable targeted delivery strategies. Nanoconjugates can be designed for controlled and stimuli-responsive release of the therapeutic agent within the tumor microenvironment (Kim and Park, 2024). These systems can also play a crucial role in overcoming mechanisms of cancer drug resistance (Yao et al., 2020), enhancing the overall drug-loading capacity (Chavda et al., 2022), and the circulation time of the therapeutic (Chaudhari et al., 2024).

A key aspect of nanoconjugates for peptide delivery in cancer is the functionalization of nanoparticles with peptide ligands that recognize specific molecules expressed or overexpressed on target cells (Raha et al., 2011), enabling selective binding and uptake of the nanoconjugate by the tumor (Kim and Park, 2024). For instance, the RGD-CendR hybrid peptide, iRGD (CRGDK/RGPD/EC), has been shown to improve penetration into tumor tissue (Chavda et al., 2022). In this regard, TASK-3 represents an attractive target, as its extracellular domains provide accessible interfaces for ligand recognition and selective targeting. In addition to targeting ligands, cell-penetrating peptides (CPPs) can be incorporated into nanoconjugate design to facilitate nanoparticle transport across cellular membranes, thereby enhancing drug delivery (Raha et al., 2011). Peptides can also serve as structural components of nanoparticles, such as in self-assembling peptide nanocarriers, or possess intrinsic cytotoxic activity that contributes directly to the therapeutic effect (Raha et al., 2011).

Various nanoparticle types are used cancer nanotherapy, including liposomes, polymeric nanoparticles, dendrimers, micellar nanoparticles, and inorganic nanomaterials such as gold nanoparticles and mesoporous silica nanoparticles (Kumar et al., 2023). Hybrid lipid-polymer nanoparticles, which combine the advantageous properties of different nanoparticle materials, as well as self-assembling peptides that serve as drug delivery vehicles are also being explored (Yao et al., 2020; Kumar et al., 2023).

The field of peptide-conjugated nanoparticles is rapidly evolving, with numerous examples of successful preclinical and clinical applications (Chavda et al., 2022). These include the delivery of cytotoxic drugs, biologics, viruses, and even other nanoparticles to tumor sites (Chavda et al., 2022). Peptide-drug conjugates (PDCs), in which a cytotoxic payload is directly linked to a targeting peptide, have demonstrated clinical efficacy, exemplified by Lu^177^-dotatate for neuroendocrine tumors and melphalan flufenamide (melflufen) for multiple myeloma (Chavda et al., 2022). Self-assembled peptide nanoparticles have been used to deliver apoptosis-inducing peptides to tumor cells (Chavda et al., 2022). Moreover, targeted delivery systems utilizing nanoparticles functionalized with peptides have demonstrated the ability to enhance drug efficacy prolong circulation times, and minimize off-target effects in the treatment of various cancers (Chaudhari et al., 2024). Although no TASK-3-targeted peptide–nanoconjugates have yet been reported, the successful targeting of this channel using monoclonal antibodies supports the feasibility of developing analogous peptide-guided delivery systems.

## 5 Rational design methodologies and algorithms for peptide-based drug development

The development of effective peptide-based drugs necessitates addressing inherent challenges, including their intrinsic flexibility, propensity for aggregation, cell permeability, enzymatic degradation, and often elusive binding affinity (Zhou et al., 2013; Farhadi and Hashemian, 2018). Rational design methodologies, leveraging computational approaches and algorithms, have become indispensable tools for accelerating the discovery of novel peptide therapeutics (Zhou et al., 2013; Farhadi and Hashemian, 2018).

*In silico* methods are employed to predict peptide affinity for their targets or binding sites and to refine design hypotheses before the experimental synthesis and testing (Zhou et al., 2013; Farhadi and Hashemian, 2018). The field of computational peptidology focuses on applying computational and theoretical methods to address peptide-related problems (Zhou et al., 2013). Artificial intelligence (AI) and machine learning algorithms are increasingly used to accelerate peptide design, screening, and optimization, thereby enabling the development of more effective peptide-based therapeutics (Nissan et al., 2024). Structure-based design uses the three-dimensional structures of target-peptide complexes, often obtained experimentally or computationally predicted, to rationally design inhibitory or activating peptides (Wang et al., 2021). For example, algorithms such as TANGO can predict peptide aggregation propensity, enabling the design of sequences with improved solubility and stability (Zhou et al., 2013; Farhadi and Hashemian, 2018). Additionally, pharmacophore modeling and virtual screening enable the identification of lead compounds by searching in structure databases for molecules that match the essential features required for target binding (Ramírez et al., 2019b).

Beyond computational tools, several design principles and strategies guide the development of peptide-based drugs. Mimicking the natural protein-protein interaction interfaces, particularly the “hot spots” that contribute most significantly to binding, is a common strategy for designing peptide inhibitors (Wang et al., 2021; Baeza et al., 2025). Stabilizing the peptide’s conformation through methods like cyclization, stapling (introducing covalent cross-links within the peptide), or incorporating non-natural amino acids can enhance its binding affinity, proteolytic stability, and cell permeability (Wang et al., 2021). Strategies to enhance cell permeability, such as conjugating the peptide to cell-penetrating motifs, are often employed (Scott et al., 2016). Avoiding amino acid sequences known to promote aggregation is another important consideration in peptide design (Zhou et al., 2013; Farhadi and Hashemian, 2018).

While the rational design of peptides targeting soluble proteins has made significant progress, designing membrane-spanning peptides that interact with ion channels such as TASK-3 poses unique challenges due to our incomplete understanding of the sequence-to-structure relationships that drive membrane insertion, assembly, and function (Niitsu et al., 2025).

## 6 Phage display technology: principles and applications in identifying peptide ligands for TASK-3

Phage display is a powerful technique that enables the identification of peptides with specific binding properties (Smith, 1985; Bazan et al., 2012). The fundamental principle of this technology is the physical link between the phenotype (peptide displayed on surface of a bacteriophage) and the genotype (DNA encoding that peptide encapsulated within the phage particle) (Smith, 1985; Bazan et al., 2012). In this technique, a library of peptides is expressed as a fusion to a bacteriophage coat protein, typically pIII or pVIII (Scott and Smith, 1990; Bazan et al., 2012). This process allows for the creation of vast libraries, containing up to 10^10^ different peptide variants (Even-Desrumeaux and Chames, 2012).

The core of phage display is the selection process, known as biopanning, where the phage library is incubated with the target protein of interest (Even-Desrumeaux and Chames, 2012). Phages displaying peptides that bind to the target with sufficient affinity are retained, while non-binding phages are washed away (Barry et al., 1996; Wu et al., 2016). The bound phages are then selected, and this selection and amplification process is typically repeated for several rounds to enrich for phages displaying high-affinity ligands (Barry et al., 1996; Even-Desrumeaux and Chames, 2012; Wu et al., 2016). Finally, the DNA from the selected phages is sequenced to identify the amino acid sequences of the displayed peptides.

Phage display technology is widely used in biomedical research and drug discovery (Cimen et al., 2025). It is used to study protein-ligand interactions, identify binding sites, and improve protein affinity for their binding partners (Cimen et al., 2025). It can also be employed to generate monoclonal antibodies and enhance their affinity, as well as to identify epitopes (Hutchings and Sato, 2024; Pohl and Almagro, 2025).

In ion channel research, phage display has proven a powerful technique for identifying inhibitors (Takacs et al., 2009; Zhao R. et al., 2015; Zhao et al., 2022; Szekér et al., 2024). Furthermore, phage display has been instrumental in identifying peptide toxins derived from natural sources or engineered by rational design that block specific ion channels with high affinity and selectivity (Takacs et al., 2009; Zhao R. et al., 2015). In some cases, phage display libraries are designed based on the known structural scaffolds of natural toxins that target ion channels, allowing the creation of focused libraries with a higher probability of yielding functional inhibitors (Takacs et al., 2009; Zhao R. et al., 2015). Modified phage display platforms are also being developed to enhance the selection of highly selective peptide binders for ion channels (Takacs et al., 2009; Zhao R. et al., 2015; Zhao et al., 2022; Szekér et al., 2024). Additionally, function-based selection strategies can be employed, in which the phage display library is screened using cell-based assays that express the target ion channel, thereby enabling direct identification of peptides that modulate channel activity (Bagriantsev et al., 2014).

While phage display is a powerful technique, it is important to be aware of potential challenges, such as the risk that certain phage clones that propagate rapidly but do not bind to the target may dominate the selection output (Hutchings and Sato, 2024; Pohl and Almagro, 2025). Therefore, careful optimization of the panning conditions and thorough validation of the identified peptide sequences are crucial for the successful identification of high-affinity binding peptides with inhibitory potential.

## 7 Integrating rational design, phage display, and electrophysiology in the development of ion channel inhibitors

Rational design can guide the creation of phage display libraries with focused diversity, increasing the likelihood of identifying high-affinity ligands for the target ion channel (Takacs et al., 2009; Zhao R. et al., 2015; Zhao et al., 2022; Szekér et al., 2024). Phage display can be used to screen these libraries and identify novel peptide sequences that bind to the target, such as TASK-3. Electrophysiology is the gold standard for validating the functional activity of identified peptide ligands (Ramírez et al., 2019b).

For TASK-3, electrophysiological validation is commonly performed in heterologous systems such as HEK293 cells or *Xenopus laevis* oocytes. Two-electrode voltage clamp in oocytes provides a robust platform for initial screening, particularly for peptide libraries, whereas mammalian cells enable detailed mechanistic and kinetic characterization. Although automated patch-clamp platforms increase throughput, their application to peptide screening remains limited by technical constraints.

Notably, the monoclonal antibody Y4, directed against the extracellular domain of TASK-3, has been shown to induce channel internalization and suppress tumor growth *in vivo* (Sun et al., 2016). This finding demonstrates that TASK-3 is accessible to extracellular targeting, particularly at the cap domain. However, no peptide ligands with comparable electrophysiological validation have been reported, underscoring a critical gap and supporting the need for peptide-based discovery strategies.

Successful examples in ion channel research illustrate the power of this integrated approach. Phage display libraries designed around the structural scaffolds of known potassium channel toxin inhibitors, such as K_V_1.3 and KcsA, have yielded novel peptide toxins with high affinity and specificity (Takacs et al., 2009; Zhao R. et al., 2015). Furthermore, the three-dimensional structures of some of these identified peptides have been determined to understand their mechanism of interaction with the ion channel (Zhao R. et al., 2015).

Applying this integrated approach to the specific challenge of TASK-3 channel inhibition for cancer therapy involves several key steps. First, rational design principles can be used to create peptide libraries based on known TASK-3 modulators, inhibitors of related K2P channels, or *de novo*-designed sequences predicted to interact with specific regions of the TASK-3 channel structure. These libraries can then be screened using phage display against purified TASK-3. The peptide ligands identified from the phage display screen would then be subjected to comprehensive electrophysiological characterization to assess their inhibitory potency, selectivity for TASK-3 over other ion channels (especially TASK-1), and their mechanism of action. Promising lead peptides can be further optimized through rational design, and the screening and validation cycle can be repeated to yield highly effective and selective TASK-3 inhibitor peptides.

## 8 Conclusion and future perspectives

Accumulating evidence indicate a substantial role of TASK-3 channels in oncogenic processes, thereby nominating TASK-3 as a plausible molecular target for future anticancer modalities. The paucity of highly selective, high-affinity TASK-3 inhibitors continues as a major bottleneck. Nevertheless, the convergence of peptide-based modalities with state-of-the-art nanoconjugate delivery platforms appears set to support precise and efficacious channel modulation.

Prioritizing rationally engineered inhibitory peptides that exhibit enhanced selectivity for TASK-3 over phylogenetically proximal channels is imperative. Collaborative integration of *in silico* design algorithms, high-throughput platforms such as phage display, and rigorous electrophysiological characterization for functional validation will be indispensable for expediting translational advancement of targeted nanconjugates against TASK-3. Defining the structural or regulatory determinants of TASK-3 that are amenable to peptide-mediated antagonism may reveal previously unrecognized therapeutic entry points. Customizing interventions based on tumor-specific TASK-3 expression profiles or mutational landscapes could, in principle, enhance the clinical impact of these precision strategies.
